# An SHA-3 Hardware Architecture against Failures Based on Hamming Codes and Triple Modular Redundancy

**DOI:** 10.3390/s22082985

**Published:** 2022-04-13

**Authors:** Alan Torres-Alvarado, Luis Alberto Morales-Rosales, Ignacio Algredo-Badillo, Francisco López-Huerta, Mariana Lobato-Báez, Juan Carlos López-Pimentel

**Affiliations:** 1 Instituto Nacional de Astrofísica, Óptica y Electrónica, Puebla 72840, Mexico; torresalv@inaoep.mx; 2 Facultad de Ingeniería Civil, CONACYT-Universidad Michoacana de San Nicolás de Hidalgo, Morelia 58000, Mexico; 3 CONACYT-Instituto Nacional de Astrofísica, Óptica y Electrónica, Puebla 72840, Mexico; 4 Facultad de Ingeniería de la Construcción y el Hábitat, Universidad Veracruzana, Maestría en Ingeniería Aplicada, Boca del Río, Veracruz 94294, Mexico; frlopez@uv.mx; 5 Instituto Tecnológico Superior de Libres, Libres, Puebla 73780, Mexico; mariana.lobato@upaep.edu.mx; 6 Facultad de Ingeniería, Universidad Panamericana, Álvaro del Portillo 49, Zapopan 45010, Mexico; clopezp@up.edu.mx

**Keywords:** SHA-3, FPGA architectures, VANET, fault tolerance, security

## Abstract

Cryptography has become one of the vital disciplines for information technology such as IoT (Internet Of Things), IIoT (Industrial Internet Of Things), I4.0 (Industry 4.0), and automotive applications. Some fundamental characteristics required for these applications are confidentiality, authentication, integrity, and nonrepudiation, which can be achieved using hash functions. A cryptographic hash function that provides a higher level of security is SHA-3. However, in real and modern applications, hardware implementations based on FPGA for hash functions are prone to errors due to noise and radiation since a change in the state of a bit can trigger a completely different hash output than the expected one, due to the avalanche effect or diffusion, meaning that modifying a single bit changes most of the desired bits of the hash; thus, it is vital to detect and correct any error during the algorithm execution. Current hardware solutions mainly seek to detect errors but not correct them (e.g., using parity checking or scrambling). To the best of our knowledge, there are no solutions that detect and correct errors for SHA-3 hardware implementations. This article presents the design and a comparative analysis of four FPGA architectures: two without fault tolerance and two with fault tolerance, which employ Hamming Codes to detect and correct faults for SHA-3 using an Encoder and a Decoder at the step-mapping functions level. Results show that the two hardware architectures with fault tolerance can detect up to a maximum of 120 and 240 errors, respectively, for every run of KECCAK-p, which is considered the worst case. Additionally, the paper provides a comparative analysis of these architectures with other works in the literature in terms of experimental results such as frequency, resources, throughput, and efficiency.

## 1. Introduction

Nowadays, cryptography has become one of the essential disciplines in information technology for data transmission and storage, since there are attacks that can compromise user security, requiring some security properties such as confidentiality, integrity, and authentication [1,2]. Confidentiality allows for determining that unauthorized users do not read data; integrity helps to know if data are not modified or altered; authentication gives certainty that some user is who they claim to be [3].

The cryptographic solutions can be implemented on software or hardware. The first ones are fixed implementations (programs), and the second ones enable exploring and researching various schemes and architectures. We can implement hardware solutions on several platforms, and the FPGA (Field-Programmable Gate Array) is one important technology that can support them. FPGAs have the capacity for reprogrammability; for this reason, a wide variety of applications use them. Reprogrammability allow for reuse or upgrades hardware designs after deployment [4].

Unfortunately, FPGAs are prone to errors in applications with several transmissions and receptions over noisy environments. These errors are called transient, pseudopermanent, and permanent [5]. Temporary errors, such as mechanical vibrations, voltage fluctuation, and particle radiation, cause transient failures. Transient errors in SRAM cells cause pseudopermanent failures. Physical damages such as manufacturing defects or gate oxide wear-out cause permanent failures. These types of failures affect FPGA devices.

For instance, Isaka, Y et al. [6] mention three types of degradation mechanisms relevant to FPGA: (i) Time-Dependent Dielectric Breakdown: an accumulation of trapped charges or defects created by a string-gate bias voltage leads to an increase in power consumption and a slowing of switching speed; (ii) Hot Carrier Injection: a collection of defects between the channel and the gate dielectric, causes an increase in threshold voltage, a decrease in carrier mobility, and slower switching; and (iii) Electromigration: a mechanism where metal ions migrate, leading to voids and deposits in interconnects, increasing trace impedance. A combination of elevated voltage and temperature accelerates these effects. Benfica et al. [7] evaluate the susceptibility of SRAM-based FPGA SEUs (Single-Event Upsets) to noise on VDD power pins and total-ionizing dose, concluding that noise on power bus pins seems more harmful to SEU cross-sections than VDD reductions. Additionally, Vargas J et al. [8] show how systems such as autonomous vehicles that use a large number of sensors can be affected by weather conditions (precipitation, fog, lightning, etc.). The sensors operate in different spectral ranges of the electromagnetic spectrum, influenced by weather and lighting conditions. Moreover, Andrew M et al. [9] generate results about classifications of cosmic rays on low, medium, and high levels. Specifically, cosmic rays induce software errors or faults at terrestrial altitudes on Earth, which is critical because FPGAs or SRAM-based devices are affected by at least one bit. The result can change and be incorrect. Furthermore, Buchner and McMorrow [10] mention that energetic particles coming from solar wind, galactic cosmic rays, and radiation belts can interact with electronics in space, avionics, and ground-based devices, causing an additional charge that alters the voltage, leading ultimately to bit upsets (a “1” goes to “0” and a “0” goes to “1”) in the FPGA.

There are different fault detection and recovery types based on physical and temporal characteristics, design, and information redundancy, such as minimum-distance coding, repetition codes, parity bits, checksum, cyclic redundant check, cryptographic hash function, Hamming Codes (HCs), and Triple-Modular Redundancy (TMR).

In this way, it is essential to highlight that hash functions are widely used to achieve integrity, authentication, and digital signatures [1,11,12]; and implemented for practical and essential solutions, e.g., blockchain [13,14,15]. A hash function, to be considered secure, needs to comply with different properties [16]: (i) unidirectionality: it is infeasible to find a message that results in a prespecified hash value; (ii) compression: some message of any length must have a digest of fixed length; (iii) easy calculation: it needs to be easy to obtain; (iv) diffusion: if only one bit of the original message is modified, the digest should flip almost half of its bits; (v) simple collision: it is infeasible to find one message which results in the same hash value as a prespecified message; and (vi) strong collision: it is computationally infeasible to find any two messages which result in the same hash value.

Within the great diversity of hash functions, the SHA-3 family is a set of hash algorithms suitable for FPGA hardware implementation since it contains simple operations, and we can use the iterative, pipeline, or unfolded approaches. Therefore, SHA-3 hardware design must reduce the impact of faults or errors to improve performance, it being necessary to detect, locate, and recover the faults at run-time [17,18].

The aim of this work is the analysis and design of techniques for presenting an iterative architecture for the SHA-3 algorithm; then, the developed architecture incorporates techniques based on data redundancy for error detection and correction. Therefore, this article presents the design and a comparative analysis of four FPGA architectures: two without fault tolerance and two with fault tolerance, which employ HCs to detect and correct faults for SHA-3 using an Encoder and a Decoder at the step-mapping functions level.

We can summarize the main contributions of the paper as follows:

- A methodology for designing and developing two architectures without fault tolerance is given. The design of the first iterative architecture implements the five step-mapping functions into the Architecture with a Single Module (ArchSM), and the second design implements the Architecture with Multiple Modules (ArchMM). We use both architectures as a platform for developing two architectures with fault tolerance.
- An architecture with fault tolerance based on HCs (ArchHC) that can detect and correct a maximum of one error for every register for a total of 120 errors for every run of KECCAK-p by using HCs formed by an Encoder and a Decoder is given.
- An architecture with fault tolerance based on HCs and TMR (ArchTMR_HC) that can detect and correct 240 errors for every run of KECCAK-p by implementing HCs to the central registers and TMR to every step-mapping function, with a voting system that determines the correct output, is given. Thus, 120 errors are detected and corrected in the central registers, and TMR masks 120 errors.
- An analysis of incremental costs is given for the four developed architectures and error coverage capacity for the two architectures with error correction and detection capacity (ArchHC and ArchTMR_HC), as well as a resources comparison among architectures without fault tolerance, with error detection and with error detection and correction. The proposed fault-tolerant hardware architecture with the highest throughput is ArchHC, whereas the one with the highest correction rate is ArchTMR_HC.
- To the best of our knowledge, no other SHA-3 architecture can detect and correct errors in the state of the art. Therefore, the coverage capacity for the architectures with error detection and recovery in the worst case is one error detection for every register. Since there are five registers, a total of five errors can be detected and corrected for every run of the Round function. Consequently, since KECCAK-p consists of 24 runs of Rounds, there are a total of 120 errors that can be detected and corrected in the worst case; if two runs are necessary, the error coverage grows to 240, and so on.

We show how our proposed architectures can be implemented in a VANET environment. In the VANET proposal system, every vehicle must contain an SHA-3 module for authentication, which serves as transceiver (transmitter and receiver) by using a message and a key for generating a message authentication code (MAC). The proposed SHA-3 fault-tolerant hardware architectures (ArchHC and ArchTMR_HC) can be part of a greater system to provide integrity, authentication, and digital certificates and part of a blockchain solution when undesirable conditions can generate and inject faults. The trade-off analysis is focused on comparing their hardware architectures’ implementations on two main ideas: space and time complexity. The ArchHC architecture provides our best throughput for the SHA-3 algorithm, which can be used on applications that require at most 276.14 Mbps, focusing on a transparent solution for the user and the system, when delivering the hash with a fault-tolerant scheme. However, ArchTMR_HC reaches the best correction rate with a higher cost of hardware resources but solves a high number of faults through TMR and HC.

The paper is structured as follows: the Section 2 presents several studies for detecting and correcting errors and faults using different FPGAs or hash functions. The Section 3 gives an overview of the SHA-3 algorithm, HCs, and TMR. The Section 4 describes our architectures and optimizations of hardware implementation. The Section 5 shows experimental results and comparisons with related works. Finally, we conclude in the last section.

## 2. Related Work

**Figure 1:**
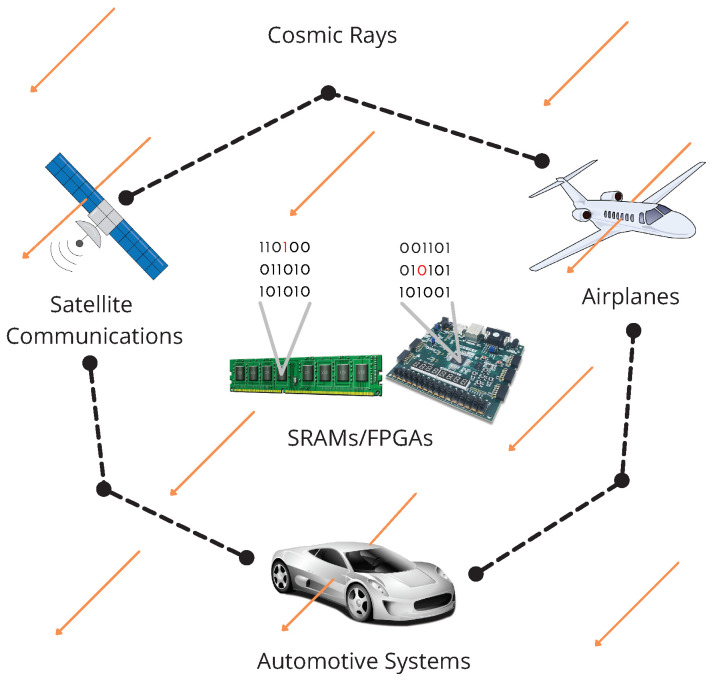
Automotive system affected by cosmic rays.

Today, systems based on processors, microprocessors, computers, microcontrollers, microcomputers, or FPGAs are critical technologies. Still, their settings or data storage can be affected by external factors such as noise, interference, weather conditions, or cosmic rays. For example, an automotive system constituted by a central computer, ECUs, and sensors can be affected by these factors, see Figure 1.

Detecting and correcting faults and errors are some of the main topics in communication, informatics, mobile, and embedded systems. There is a growing population of devices coexisting in an environment of distinct types of communication networks. Previously, other works proposed solutions for resolving redundancy schemes for the SHA-2 algorithm, which has not presented security problems yet. Nevertheless, the SHA-3 proposal offers a modern and exciting alternative to provide security services such as authentication, integrity, digital certificates, blockchain, etc. In this sense, it is necessary to evaluate redundancy solutions and implement them for our proposal. To the best of our knowledge, existing SHA-3 architectures mainly search to detect errors but not to correct them; thus, works based on parity checking and properties of the algorithm can be found in the state of the art. The research of Frank H. [19] presents additional considerations and design techniques employed with an SRAM-based FPGA vulnerable to radiation-induced errors (this situation does not occur with nonreconfigurable devices). These devices are used in a space-based processing system to achieve high operational reliability. However, these considerations and traditional techniques—such as configuration bitstream scrubbing, TMR, error-correcting codes (ECC), user memory protection, and combined mitigation approaches—can be used in terrestrial applications. Solutions based on replicating modules require many hardware resources, low performance, and high power consumption.

Luo et al. [20] implemented a parity-checking-based error detection method for the SHA-3. A *cryptographic* module, which computes every operation at the step-mapping level, is helped by another module called the *protector* composed of three parts: the *predictor*, *compressor*, and *comparator*. Each piece fulfills one different necessity: the *predictor* reads the input, and the *compressor* reads the output after the transformations are made by the *cryptographic* module; then, the *comparator* computes the results of the *predictor* and *compressor*; if a mismatch occurs, an alarm is activated, and the error is detected. The authors say that the system achieved a correct detection of 83.60% of injected faults.

Bayat-Sarmadi et al. [21] made use of one property of the state array for the SHA-3 algorithm, where a random number rotates a lane before the step-mapping operations, and then it is shifted back after operations. Two modules are implemented, one with this property and another without modifications, the results are compared, and, if any difference exists, the error can be detected. The authors mentioned that the proposed method could detect 100% of multiple random fault injections.

Additionally, Juliato and Gebotys [22] proposed five different schemes for the SHA-256: (i) full TMR, where the circuit is triplicated and a voting system is used to determine the output; (ii) in TMR with shared encoded memory, the three SHA-256 modules share the constant memory, HCs protect it, and then a voter system determines the output; (iii) the TMR for registers and shared encoded memory scheme moves the TMR at the register level and uses an encoded shared memory to protect the constants; (iv) in HCs for all registers, an Encoder and Decoder are used in every register before any write and read operation; and, finally, in (v) HCs for main registers, only the registers involved in some operation are protected using Encoders and Decoders. The paper concludes that their proposed fault tolerance scheme can be used in applications that require lower consumption, besides error correction and detection.

In the work proposed by Michail H. et al. [23], two totally self-checking (TSC) devices were implemented for the SHA-1 and SHA-256 algorithms. The TSC device is formed by: (i) the Functional Circuit Module, which is composed of the Information Symbol Generator and the Check Symbol Predictor, and (ii) the Checker Circuitry, which includes the Check Symbol Complement Generator and an *r*-bit Two-Rail Checker. The results shown by the authors demonstrate that TSCs can detect 100% of odd faulty bits and that it is more efficient in terms of area, throughput/area, and power consumption than Duplicated with Checking architectures.

In Table 1, we compare related works focused on secure hashing algorithms such as SHA-1, SHA-2, and SHA-3. The works for SHA-3 present architectures that only have detection capacities, different from our proposed architecture that can detect and correct errors. We analyze the design techniques used for related work for the SHA-2 algorithm, proposing two hardware architectures without tolerance and two architectures with tolerance. Hence, the last two SHA-3 hardware architectures take into account: (i) a structure with fault tolerance that uses HCs in the central registers to detect and recover errors, implementing an Encoder and a Decoder, and (ii) a structure implementing HCs and a TMR in every step-mapping function, allowing for continued operation in the presence of errors.

## 3. Preliminaries

The propagation model in a VANET must consider the effects of potential interference of wireless communication from other vehicles and the existence of largely deployed access points [24]. In VANETs, the propagation model operates in three environments: highway, city, and rural. The propagation model is usually assumed to be a free space on a highway; nevertheless, the reflection of the wall panels around the roads affects the signal. In a city, communication is complex due to the variable vehicle density, buildings, trees, and obstacles to signal propagation; such obstacles cause shadowing, multipath, and fading effects. In rural environments, communication is affected due to complex topography; hence, it is important to consider the signal reflection and the attenuation of the signal propagation.

In a VANET, we can integrate devices such as Commercial Reconfigurable Processors [25], Systems on Chip integrating FPGA [26,27,28,29], and FPGAs that have been integrating processing cores for years as On-Board Systems. These devices present several problems that can affect some change in the configurations or calculations stored in memories or modules within the FPGA such as cosmic rays, noise effects such as temperature and sounds, alterations by a read or write to another cell, and so on [5,6,7,8,18]. These problems can change one bit from logic level 1 to logic level 0 or vice versa, and FPGAs are mainly based on RAM, which provides many advantages, but in real situations, outdoor applications, and environments with many devices and communication networks (originated by the growth of the IoT, IIoT, and Industry 4.0), these problems rise. On the one hand and in certain applications, changing one bit is not a big problem, for example, changing one bit in an audio, image, or video can generate imperceptible results, and this is originated because they are sent as plain text and one bit does not provide great information, according to Shannon’s fundamental theorems. On the other hand, this situation becomes critical when we want to send secure messages, especially with encrypted solutions, where a single change generates completely incomprehensible results, due to the high diffusion that cryptographic algorithms provoke in their data processing and to the great amount of information that each bit provides.

Therefore, in data transmission, no system can prevent errors caused by natural or human phenomena, such as noise originated by electronic devices or radiation. However, some techniques based on redundancy help detect whether the information received is the same as the original data transmitted. Redundancy is the repetition of hardware or information to increase the system’s reliability in which the cost and complexity increase. Nevertheless, it is a rule to follow if we require a robust design for operating in environments that cause errors. There are several techniques for improving data communications; specifically, some existing redundancy techniques are HCs and TMR.

HCs present an improvement compared to codes based on parity bits, since the latter can find errors in one bit but not correct them. TMR, in contrast to HCs, has the advantage of detecting errors and correcting them. TMR, as the name implies, provides three replicas of the same module, which has the advantage that if one fails, the other two can mask the fault and continue operating correctly, increasing the system’s robustness.

In the following subsections, we describe two main ideas: (1) the hash functions and the operations involved in developing the SHA-3 algorithm such as step-mapping, Round, KECCAK, KECCAK-p, and Sponge; and (2) the redundancy techniques for error detection and correction based on HCs and TMR, which allow the hardware architecture for the SHA-3 algorithm to robustify and strengthen.

### 3.1. Hash Function

Several hash algorithms have been developed to assure security properties. For instance, the MD5 algorithm extends the MD4 message-digest algorithm [30]; MD5 takes a message of an arbitrary length and produces an output of 128 bits. However, the MD5 algorithm is now broken since it suffers from extensive vulnerabilities. Another algorithm is SHA-1, which was designed by the United States National Security Agency [31] and takes a message of an arbitrary length less than $2^{64}$ and produces an output of 160 bits [32]. RIPEMD is a family of cryptographic hash functions called RIPEMD, RIPEMD-128, RIPEMD-160, RIPEMD-256, and RIPEMD-320, with RIPEMD-160 being the most common; however, RIPEMD and RIPEMD-128 are no longer considered secure, and RIPEMD-160 is about 15% slower than SHA-1 [33].

In 2011, NIST formally deprecated SHA-1; then, SHA-2 was adopted, a set of cryptographic hash functions consisting of six elements called SHA-224, SHA-256 SHA-384, SHA-512, SHA-512/224, and SHA-512/256 [34]. The SHA-2 algorithm follows the same structure of message expansion and iterates state update transformation, as SHA-1. Nonetheless, since the design still shows significant similarities with the SHA-1 hash algorithms, it is not unlikely that vulnerabilities will be found in the (near) future [35].

One essential hash function is called Secure Hash Algorithm 3 (best known as SHA-3). SHA-3 is a hash function family that consists of four fixed-length functions called SHA3-224, SHA3-256, SHA3-384, and SHA3-512 and two extensible functions called SHAKE-128 and SHAKE-256 [36], which are based on an algorithm called KECCAK. We remark that KECCAK was selected winner by the NIST (National Institute of Standards and Technology) in the SHA-3 Cryptographic Hash Algorithm Competition [37,38]. SHA-3 has several applications, such as generation and verification of digital signatures, key derivation, and pseudorandom bit generation. In addition, SHA-3 has advantages for design and security, allowing for flexibility in the implementation [39].

For instance, the KECCAK sponge function should stand by its security claim even if the number of Rounds is divided by two; the sponge function is provably secure against generic attacks; unlike SHA-1 and SHA-2, SHA-3 does not have the length-extension weakness and hence does not need the HMAC nested construction. SHA-3 can be natively used for hashing, full-domain hashing, randomized hashing, stream encryption, MAC computation, and tree hashing. The instances for SHA-3 and SHAKE make use of a single permutation for all security strengths, cutting down implementation costs compared to hash function families by making use of two (or more) primitives, such as the SHA-2 family. Additionally, SHA-3 excels in hardware performance and has overall good software performance.

### 3.2. SHA-3 Algorithm

The SHA-3 algorithm is defined for a digest length *d* with size 224, 256, 384, or 512 and a message *M* with two bits “01" added at the end, such that *SHA*3−d(M)=KECCAK[c](M||01,d), where *SHA3* and KECCAK are functions, *M* is the input string to the SHA-3 algorithm, and the operator || indicates concatenation [36]. KECCAK is a family of sponge functions that are parameterized for any choice of *r* and *c*, where r+c=b and *b = {25, 50, 100, 200, 400, 800, 1600}*. *KECCAK* is described by the use of the SPONGE function, such that KECCAK[c](N,d)$=SPONGE\left[KECCAK-P\left[b,n_{r}\right],pad10^{*}1,r\right]\left(N,d\right)$ where N=M||01 is the input string to functions SPONGE or KECCAK. In the SPONGE function, an arbitrary number of bits are absorbed into the state of the function, and an arbitrary number of bits are squeezed out of its state. The sponge function shown in Algorithm 1 receives different arguments: the padding function *pad*, the KECCAK-p function *f* (see Algorithm 2), the positive constants *r* and *c*, the message *N*, and the size of the hash *d*.

The padding function *pad* receives the positive integer *r* and a non-negative integer m=len(N) as inputs. The output is a string *P* such that $P=1||0^{j}\left|\right|1$, where *j* is obtained by j=(−m−2)mod *r*. The *KECCAK-p* algorithm consists of 24 permutations of the Round function for the vector *S* of length b=1600. Round is formed by five step-mapping functions called θ, ρ, π, χ, and ι (see Algorithm 3): (i) in function θ, each bit in the state array is operated with the parity of two columns; (ii) in function ρ, the bits of each lane are rotated by a length called *offset*, the value *t* varies from 0 to 23, and in each iteration, the *x* and *y* values take *y* and (2x+3y)mod5 values, respectively; (iii) in function π, lane positions are rearranged; (iv) in function χ, each bit of a row is XOR-ed with the result of a nonlinear function of two other bits of the same row; and (v) in function ι, some bits in Lane(0, 0) are modified by the Round constants *RC*, see Table 2.
**Algorithm 1:** SPONGE Algorithm
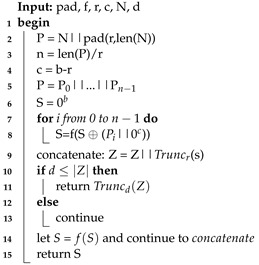

**Algorithm 2:** KECCAK-p Algorithm
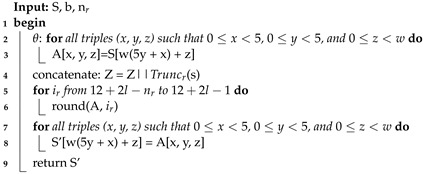

**Algorithm 3:** ROUND Algorithm
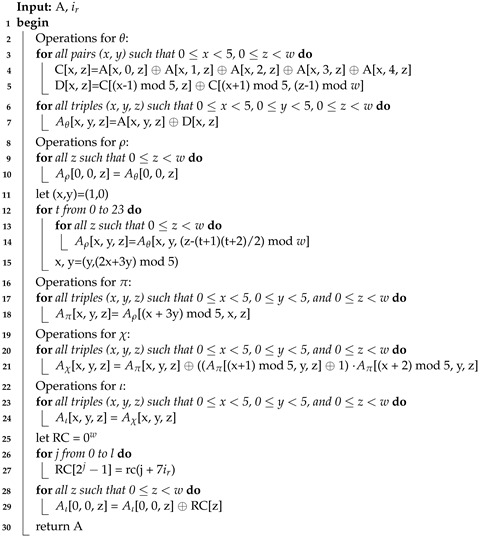



Additionally, there are two SHA-3 extendable output functions called SHAKE-128 and SHAKE-256, which are defined from the KECCAK[c] by appending a four-bit suffix to the message M and for any output length d such that SHAKE128(M,d) = KECCAK[256](M||1111,d) and SHAKE256(M,d) = KECCAK[512](M||1111,d).

### 3.3. Hamming Codes

**Figure 2:**
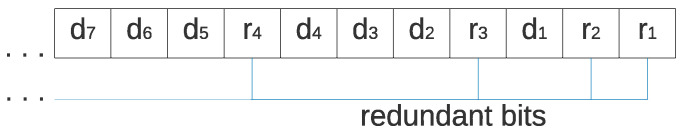
Codeword.

HCs are a class of linear codes [40] invented in 1950 by Richard Hamming [41] to detect and correct errors. The errors can be detected and corrected by adding *m* redundant bits to a binary message of length $2^{m}-m-1$, forming a code word of $2^{m}-1$ bits. The redundant bits are placed in powers of two, while the data bits of the message are placed in the empty spaces; thus, $r_{i}$ corresponds to the redundant bits and $d_{i}$ corresponds to the message such that i∈N, as is shown in Figure 2.

The redundant bits used by HCs are parity bits, which can be of two different types: even and odd. For even parity bits, the number of *ones* is counted in a set of bits; if the count is odd, the parity bit takes the value of *one*, and if the count is already even, the parity bit takes the value of zero. For odd parity bits, if the count of ones is even, the parity bit takes a value of *one*, and if the count is odd, the parity bit value is *zero*. Counting is conducted with an XOR operation, either for odd or even parity. The codeword is received for error detection and correction (containing the redundant and data bits). The HCs Algorithm calculates $b_{n}$ check bits (one for every redundant bit) by XOR-ing all bits whose binary representations have a one in the *i*-th least significant bit for the *i*-th check bit. The check bits are placed from the $b_{1}$ check bit in the least significant position to the $b_{n}$ check bit in the most significant position, such that $e=b_{n}b_{n-1}b_{n-2}\ldots b_{1}$; therefore, it is possible to obtain a value that indicates the position of the error. If there is no error, all check bits will have a value of *zero*; in contrast, if a value different than zero is obtained, there is an error in the position of the obtained value; thus, a bit-flip in that position is made for correcting the error.

### 3.4. Triple-Modular Redundancy

In TMR, three replicas of a component run in parallel and a majority voting system processes the result to produce a single output [42]. The circuit of the voting system is composed of three AND gates (operator ·) and two OR gates (operator +), as shown in Equation (1).

(1) $$output=i_{1}\cdot i_{2}+i_{1}\cdot i_{3}+i_{2}\cdot i_{3}$$

where $i_{j}$ with *j* = 1, 2, and 3 are the inputs to the system, analogously, the voting system can be seen as an if statement, with four possible outputs: (1) if $i_{1}$ is equal to $i_{2}$ and $i_{3}$ is different, then $i_{1}$ (or $i_{2}$, since they have the same value) is the obtained output; (2) if $i_{1}$ is equal to $i_{3}$ and $i_{2}$ is different, the output is $i_{1}$ (or $i_{3}$); (3) if $i_{2}$ is equal to $i_{3}$ and $i_{1}$ is different, in this case, the obtained output is $i_{2}$ (or $i_{3}$); and (4) if the three inputs are different, in this case, a correct output cannot be determined.

The logic equation represents the behavior of TMR using logic gates; however, the final design depends on the tool used for synthesis and implementation and the type of programming. In this case, TMR was created using if-else instructions in Vivado 2020.1 (a tool for developing VHDL code), resulting in a combination of 3217 LUTs and 1602 FFs. Using other tools and a different type of implementation may give different results.

## 4. Methods

In the VANET paradigm, there is an exchange of safety (road accident, roadblock, accident information, etc.) and nonsafety communication (tolling information or entertainment) among vehicles. For vehicle-to-vehicle (V2V), the communication is made using a multihop technique, as long as the vehicles are in the transmission range of each other, in contrast to V2I communication, where the communication is made also using the multihop technique with the help of roadside infrastructure such as roadside units (RSU) [43,44]. These transmitted messages among vehicles need some calculation made by computers in order to evaluate different characteristics: For providing sufficient quality of service in V2V communication, it is necessary to compute how far the message propagates and how long it takes to deliver the message to the vehicles to help the drivers to make appropriate decisions on time; thus, the analysis of transient behavior is crucial in many safety scenarios [45]. For congestion protocols for VANETs, there are probabilistic model-checking techniques in order to analyze uncertain and unpredictable behaviors [46]. Salvador Gonzalez and Victor Ramos [47] studied the loss process of broadcast packets over the control channel in VANETs; they said that for most of the messages, an increase in packet delivery time does not have a significant impact on network performance. On the contrary, it is very important that messages arrive correctly; however, for safety and critical messages, the packet delivery time and consequently the loss rate have great importance. In contrast, hardware elements that contain the data to be transmitted are susceptible to noise and radiation, which may affect one or more bits inside registers or affect the functioning of some LUTs by voltage oscillation, for example, which is a huge problem because they are used in the SHA-3 function, resulting in a gap in security, since a completely different result can be obtained.

Every vehicle contains one SHA-3 module for authentication, which serves as sender and receiver by using a message and a key for generating a message authentication code (MAC). A vehicle in the VANET network sends the generated MAC along with the message to another vehicle in the network, which uses the SHA-3 algorithm for generating another MAC, using the received message and its key; then, the receiver compares its MAC to the MAC of the sender, and if they are equal, the message is authenticated. In the opposite case, the message can be rejected.

Designing and developing fault and error detection architectures requires us to know and evaluate the different processes that are carried out and defined by the SHA-3 algorithm, so the general methodology is described in four steps:

1. Analysis and design of forwarding iterative architectures without error detection and correction;
2. Design and implementation of a hardware architecture for detecting and correcting based on HCs;
3. Analysis and improvement of the previous architecture for developing a new architecture for fault tolerance;
4. Comparison among proposed architectures and comparison against related works.

Following these steps, four architectures are developed and analyzed:

- An integral architecture for SHA-3 without fault detection (ArchSM);
- A modular architecture for SHA-3 without fault detection (ArchMM);
- An SHA-3 architecture for error detection and correction using HCs (ArchHC);
- An SHA-3 architecture using HCs and TMR (ArchTMR_HC).

It is important to emphasize that our methodology offers an additional advantage related to the possibility of directly and fairly comparing the two architectures without error detection and correction against the two architectures with detection and correction of errors. Hence, we can compare the hardware design between (1) the base architectures (without tolerance) and (2) the modified architectures (with tolerance). This comparison provides an adequate reference measurement and an evaluation of what characteristics are obtained and lost, such as a trade-off in terms of latency, LUT, FF, minimum period, maximal frequency, throughput, and efficiency.

**Figure 3:**
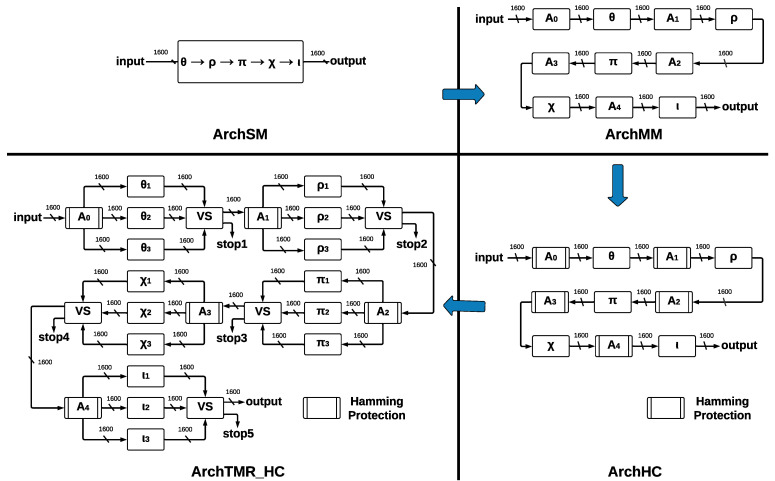
Development of architectures from ArchSM to ArchTMR_HC.

The process for providing the final architecture ArchTMR_HC is shown in Figure 3, where the four developed SHA-3 hardware architectures present different modules and report individual implementation results.

The proposed architectures, see Figure 3, use an FPGA VIRTEX 7 and Vivado 2020.1 (a tool for developing VHDL code) as platforms to carry out the tasks of design, development, analysis, and testing. However, we remark that the four proposed architectures have a generic approach allowing their implementation on several FPGA platforms (Virtex, Spartan, Cyclone, Stratix, Arria, Certus, and CrossLink, among others) and ASICs (application-specific integrated circuits). Our proposed designs do not specialize in taking advantage of specific resources (LUTs, flip-flops, array structure, slices, DSPs, chip technology, etc.) of any FPGA technology by some manufacturer such as Xilinx, Altera, Lattice, Actel, etc. In addition, the architectures can behave better or worse in different FPGA technologies, depending on their internal structures, their hardware resources, algorithms, and the Place/Route strategies of the implementation tools. This fact requires other research topics to explore different FPGAs and which ones can deliver better results.

The basic architecture is called ArchSM, where we implement the five step-mapping functions in a single module. If some error occurs in ArchSM, the hash in the output will have a completely different result. Then, we implement the five step-mapping functions separately, forming five separate modules and five central registers. This new architecture is called ArchMM; however, now the errors can occur in the register and the step-mapping functions, which leads to the development of a new architecture. Implementing HCs in the registers that store the vector makes it possible to recover the original vector if one error occurs. This architecture is called ArchHC; nevertheless, if one error occurs in some step-mapping function, the hash will be different than the expected one since they have no protection.

To protect step-mapping functions against errors, we implement the TMR to each one. If one error occurs in some module of some step-mapping function, the voting system ignores such a fault. The correct vector will pass to the following step-mapping function; nevertheless, if the three inputs are different, we cannot obtain a correct output, and a *stop* flag is activated. By combining TMR and HCs, we construct the final architecture, called ArchTMR_HC.

The sponge function is the same for all architectures; the state machine in all architectures is centered at the KECCAK-p level since step-mapping functions are implicit. For notation in the state machine, the *ok* suffix is equivalent to *READY*; thus, *ok* indicates when some function has been completed; for example, if $\theta_{ok}=1$, then θ output is ready to be communicated to the next module.

### 4.1. System Model

**Figure 4:**
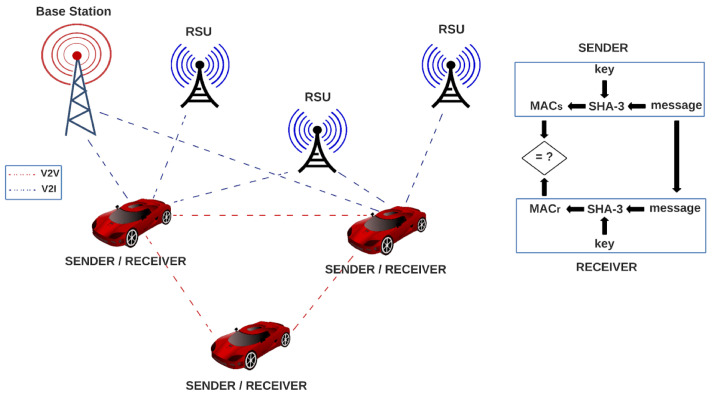
System model to implement SHA-3 architectures in VANETs.

In Figure 4, we show how our proposed architectures can be implemented in a VANET environment. In the system, every vehicle contains one SHA-3 module for authentication, which serves as sender and receiver by using a message and a key for generating a message authentication code (MAC). A vehicle in the VANET network sends the generated MAC along with the message to another vehicle in the network, which uses the SHA-3 algorithm for generating another MAC, using the received message and its key. Then, the receiver compares its MAC to the MAC of the sender, and if they are equal, the message is authenticated. In the opposite case, the message can be rejected.

### 4.2. SHA-3 Architecture

**Figure 5:**
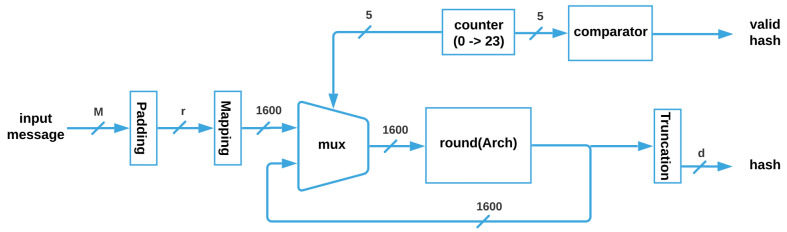
SHA-3 general architecture.

The SHA-3 architecture, shown in Figure 5, receives the input message M, whose maximum size is 1600−2×d; then, it is padded for getting a vector of size *r*, which is mapped to obtain the 1600 bits that enter through the multiplexer to the round Archi function, (where i can be SM, MM, HC, or TMR_HC). Next, the vector is feeded back until the 24 Rounds are completed, and when the counter is equal to 24, a flag is activated through a comparator, and the correct truncated hash with size *d* is obtained.

Round(Arch) is a function where the parameter Arch can be substituted for any of the four architectures without (ArchSM and ArchMM) and with (ArchHC and ArchTMR_HC) fault tolerance that are described next.

### 4.3. SHA-3 Architecture without Fault Tolerance

We propose two SHA-3 hardware architectures without fault tolerance: the first architecture is called ArchSM (Architecture Single-Module) and implements five step-mapping functions in a single module. The second architecture is called ArchMM (Architecture Multi-Module) and implements five step-mapping functions as different modules.

#### 4.3.1. ArchSM

**Figure 6:**
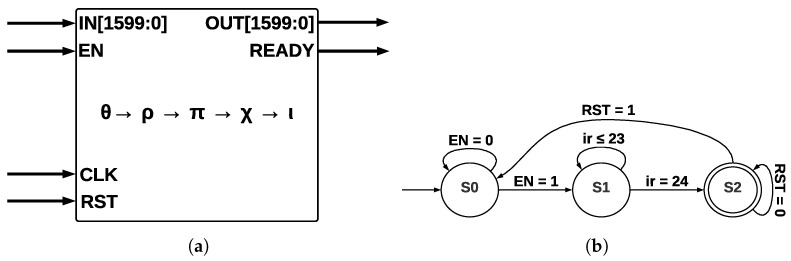
ArchSM at Round level and ArchSM state machine at KECCAK-p level. (**a**) ArchSM at Round level. (**b**) ArchSM state machine at KECCAK-p level.

The five step-mapping functions (θ,ρ,π,χ,ι) were implemented in the same module (see Figure 6a), which has four inputs—*IN*, enable *(EN)*, clock *(CLK)*, and reset *(RST)*—and two outputs—*OUT* and a control signal called *READY* that pinpoints when the process has been completed.

The input *IN* is operated by five step-mapping functions; then, bus *OUT* is obtained; Round constants are stored inside the module as arrays and operated on directly in ι. After 24 runs of Round, the state machine (see Figure 6b) indicates that KECCAK-p function has been completed. The process starts in state S0; when the *EN* signal takes a value of 1, a transition to state *S1* is made, and then 24 runs of *Round* function are counted. When this counter takes a value of 24, the process has been completed; in state *S2*, signal *RST* allows a new run of KECCAK-p.

#### 4.3.2. ArchMM

**Figure 7:**
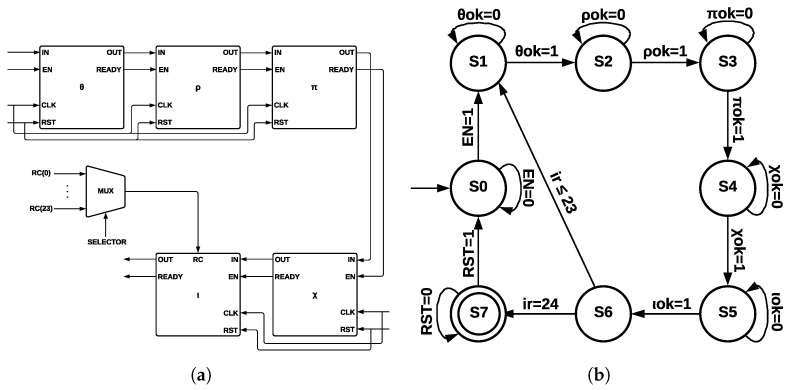
ArchMM at Round level and ArchMM state machine at KECCAK-p level. (**a**) ArchMM at Round level. (**b**) ArchMM state machine at KECCAK-p level.

Each one of the five step-mapping functions was implemented as a different module (see Figure 7a). Each module has four inputs—*IN*, enable *(EN)*, clock *(CLK)*, and reset *(RST)*—and two outputs—*OUT* and a control signal called *READY*, which indicates when the process has been completed. The output of θ and its *READY* signal are connected to ρ input and to its *EN* signal since ρ is activated when θ has been completed; the process is repeated for the remaining functions until ι output is obtained.

Round constants are stored as arrays, and they are multiplexed into ι by a control signal of five bits, corresponding to variable $i_{r}$. Figure 7b shows the ArchMM state machine, which introduced extra states since step-mapping functions were implemented as separate modules. There are eight states in the ArchMM state machine, going from S0 to S7; the initial state corresponds to S0 and the final state to S7. The module θ is represented by S1, ρ by S2, π by S3, χ by S4, and ι by S5. State S6 controls if 24 runs of Round have been completed. The Round constants are disclosed by cryptographic systems’ definition and also the algorithm used with all its internal processes. The algorithm’s strength lies in the computational complexity of the direct and inverse mathematical calculations.

### 4.4. SHA-3 Architecture with Fault Tolerance

We propose two SHA-3 hardware architectures with fault tolerance. The first is called ArchHC, which uses HCs, implementing two extra modules: a Hamming Encoder and a Hamming Decoder. The second is called ArchTMR_HC, which uses HCs and TMR.

#### 4.4.1. ArchHC

The five step-mapping functions implemented as modules in ArchMM were reused in ArchHC along with two extra modules: Hamming Encoder and Hamming Decoder.

Hamming Encoder: The input length is 1600; thus, eleven redundant bits are necessary. Hamming Encoder calculates the position of eleven redundant bits and then calculates their values. These positions are $2^{0},2^{1},2^{2},2^{3},2^{4},2^{5},2^{6},2^{7},2^{8},2^{9},$ and $2^{10}$ for a total of 1611 bits; thus, the output size is 1611; each value of the redundant bits is calculated using even parity and an XOR operation over every bit whose value in $i_{th}$ position is equal to one for the $i_{th}$ redundant bit. Hamming Encoder has four inputs—IN, enable *(EN)*, clock *(CLK)*, and reset *(RST)*—and two outputs—*OUT* and a control signal called *READY*.

**Figure 8:**
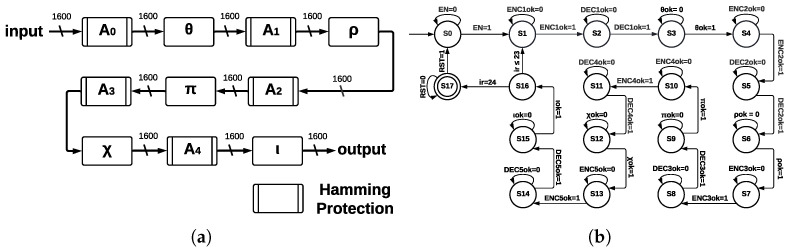
ArchHC at Round level and ArchHC state machine at KECCAK-p level. (**a**) ArchHC at Round level. (**b**) ArchHC state machine at KECCAK-p level.

Hamming Decoder: It is implemented before every step-mapping function and detects one error in any bit using parity check, which allows for detecting incorrect bit position, and finally, a gate not corrects this error. Hamming Decoder has four inputs—coded signal (*IN*), *EN*, *CLK*, and *RST*—and two outputs—decoded signal *OUT* and a valid output indicator (*READY*). ArchHC is shown in Figure 8a; Hamming Encoders and Hamming Decoders protect every register against faults (Hamming protection) since errors can be detected and corrected. The registers are named $A_{0}$, $A_{1}$, $A_{2}$, $A_{3}$, and $A_{4}$; these registers are the inputs to the step-mapping functions θ, ρ, π, χ, and ι, respectively.

The control process for ArchCH is shown in the state machine of Figure 8b. In addition to states representing step-mapping functions, extra states are necessary since we introduce Hamming Encoders and Hamming Decoders.

The state machine of ArchHC has 18 states, going from S0 to S17; the initial state is S0, the final state is S17, and the counter for determining 24 runs of Round corresponds to state S16. States S3, S6, S9, S12, and S15 correspond to step-mapping functions, whereas the remaining states represent Hamming Encoders and Hamming Decoders.

#### 4.4.2. ArchTMR_HC

**Figure 9:**
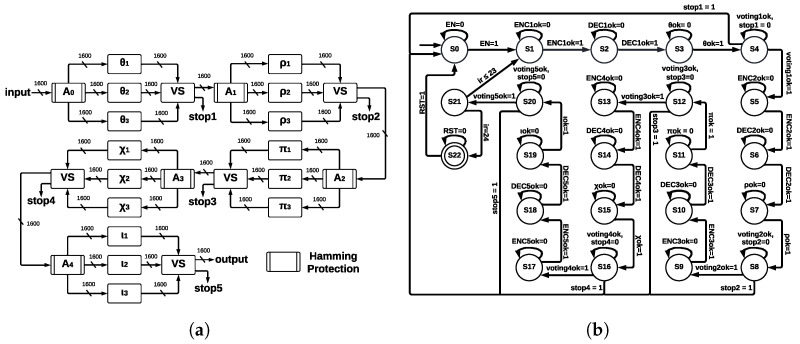
ArchTMR_HC at Round level and ArchTMR_HC state machine at KECCAK-p level. (**a**) ArchTMR_HC at Round level. (**b**) ArchTMR_HC state machine at KECCAK-p level.

This architecture implements TMR in every step-mapping module, and then a voting system (VS) determines the winner output. The five central registers are protected by Hamming Encoders and Hamming Decoders, which allow for error detection and correction capacity; ArchTMR_HC architecture is shown in Figure 9a.

The input is stored into $A_{0}$; then, it is sent to θ, which is triplicated (TMR). The process in each module θ is executed, and we obtain three outputs. These outputs enter the voting system, where a combinational circuit is executed and a single winner output is obtained, which is stored in $A_{1}$. If the three inputs are different, a correct result cannot be obtained; thus, a flag indicates the error, and the algorithm stops. This process is repeated for the remaining registers and step-mapping modules until an output of the final voting system (connected to ι) is obtained. Hamming protection (formed by Hamming Encoders and Hamming Decoders) allows for detecting and correcting errors at the register level. In contrast, TMR applied at step-mapping allows for continuing operating correctly in the presence of errors. We show the state machine for ArchTMR_HC in Figure 9b; in addition to states of step-mapping modules and Hamming Encoders and Decoders, extra states for the voting systems are introduced. The state machine is formed by 23 states, from S0 to S22; the initial state is S0, and the final state is S22; state S21 controls 24 runs of Round. Step-mapping modules correspond to states S3, S7, S11, S15, and S19; voting systems to states S4, S8, S12, S16, and S20; and remaining states correspond to Hamming Encoders and Hamming Decoders.

The Hamming Encoding is applied after the voting system since the winner output needs to be protected before sending it to the next step-mapping function. If there is an error in this stage, we identify three possible faults for ArchTMR_HC: (1) errors in one module, (2) errors in two modules, and (3) errors in three modules. Each voting system stage has a “stop” signal, which is activated if there are errors in at least two of the three modules (points 2 and 3). In this case, it cannot be determined which output is correct since this is the nature of the TMR approach. The voting system is implemented as if-else instructions. Thus, the resources needed for its development depend on the instrument used and the type of codification; in our case, Vivado 2020.1 requires 3217 LUTs and 1602 FFs. We can infer that the synthesis is made bit by bit. Finally, it could be any number of faults in one module; this will result in one vector of 1600 bits different from the other two vectors; then, the majority voting system will ignore the faulty vector, and the correct one will be transmitted to the next module.

Next, these four architectures are evaluated, making fair comparisons among our designs, which provide analysis as references, as well as making comparisons with work related to both fault-tolerant and nontolerant schemes.

## 5. Results

This section provides results for the developed SHA-3 architectures such as resources comparison, incremental costs, and error coverage capacity.

### 5.1. Resources Comparison for SHA-3 Architectures with and without Fault Tolerance

When algorithms are implemented on some platform, we might computationally compare their implementations on two main ideas: time and space complexity. The first quantifies the time to execute as a function, and the second quantifies the required amount of space or memory. In this work, hardware implementations are developed, tested, and compared. The parameters used for comparing are:

- Time: latency, minimum period, and clock frequency.
- Space: look-up tables, flip-flops, and hash size.
- Time and space: throughput and efficiency rates for combining both parameters.

In the end, comparisons on hardware can be unfair if different technologies are used, but the implementation results enable us to provide values as references, see Table 3.

A fair comparison is challenging. It depends on each technology, and the same design can represent benefits by the FPGA of one manufacturer and be penalized by the FPGA of another one or other technology such as ASIC. Therefore, we present the trade-off analysis, highlighting that (1) we compare our fault-tolerant designs against fault-tolerant related work as black boxes since a fair comparison is complicated (e.g., different goals, platforms, and results); (2) trying to solve the above, we propose our nontolerant architecture to compare with our tolerant architecture and have a fair comparison with implementation results that give a reference in hardware architectures in a better way; and (3) we present our nontolerant architecture against nontolerant related works to show that the design is competitive.

Results for the two architectures without fault tolerance (ArchSM and ArchMM) and for the two architectures with fault tolerance (ArchHC and ArchTMR_HC) are shown in Table 3.

For architectures without fault tolerance, we show in Table 3 that ArchSM achieved a latency of 27 clock cycles in all four hash sizes, a maximum throughput of 13 852 Mbps in the hash size 384, and maximum efficiency of 5.90 Mbps/LUT, also in the hash size 384, whereas ArchMM achieved a latency of 199 clock cycles in the four hash sizes, a maximum throughput of 804 Mbps in the hash size 256, and maximum efficiency of 0.27 Mbps/LUT in the hash size 256. These results show that ArchSM is superior in all evaluation metrics.

For architectures with fault tolerance, we show in Table 3 that ArchHC had a latency of 299 clock cycles in the four hash sizes, a maximum throughput of 276 Mbps in the hash size 512, and maximum efficiency of 0.01 Mbps/LUT, also in the hash size 512. In the case of ArchTMR_HC, the achieved latency in the four hash sizes is 443, the maximum throughput was obtained by the hash size 384 with 230 Mbps, and the maximum efficiency was obtained by the hash size 512 with 0.008 Mbps/LUT. ArchHC reports better results in most evaluation metrics, and the determination of the better architecture with fault tolerance depends on the number of errors that the architecture can detect and correct. We show and discuss these results in the Error Coverage Capacity for SHA-3 Architectures with Fault Tolerance section.

### 5.2. Example of Detection and Correction of Errors

An example of how the errors can be detected and corrected is shown in Table 4, consisting of seven different steps, taking the first seven bits of a total of 1600 and the theta step-mapping function. The process can be extended to any function and the 11 redundant bits necessary for covering the 1600 bits. (1) The first seven bits (called *m*) of a total of 1600 are taken. (2) The XOR operation calculates the *i*-th parity bit whose binary representation has a one in the *i*-th least significant bit, in this case, $p_{1}$, $p_{2}$, $p_{3}$, and $p_{4}$. They are added to the message *m* for forming a message $m^{\prime }$; this process is shown in Table 5a. (3) One error is generated in a random bit; in this case, the bit at position seven is changed from one to zero. (4) The error is detected and corrected when the data are transmitted through the Hamming Decoder by using four parity check bits ($pc_{1}$, $pc_{2}$, $pc_{3}$, and $pc_{4}$), which determine the position of the error; in this case, the obtained value is seven (0111); this process is shown in Table 5b. The bit at the seventh position is changed using a not instruction. (5) After correcting the error, the original message is recovered and is transmitted to the three theta modules ($\theta_{1}$, $\theta_{2}$, and $\theta_{3}$). (6) Errors are generated in a random module, for example, in $\theta_{1}$, which causes a different output than the outputs of $\theta_{2}$ and $\theta_{3}$. (7) The outputs of the three theta modules are the inputs to the voting system that determines the output through a majority vote.

Letter $d_{i}$ represents the data with *i* = 1, 2, 3, 4, 5, 6, 7, and $p_{j}$ represents the parity bits with *j* = 1, 2, 3, 4, in the Hamming Encoder process (see Table 5a). For calculating $p_{1}$, the data bits whose binary representations have a one in the first position are XOR-ed. The data bits for calculating $p_{1}$ are $d_{1}$, $d_{2}$, $d_{4}$, $d_{5}$, and $d_{7}$. For calculating $p_{2}$, the data bits whose binary representations have a one in the second position are XOR-ed. The same idea is applied for calculating the remaining parity bits. Table 5b shows the process for detecting and correcting the injected error (bit $d_{4}$ was changed from one to zero), where four parity check bits (pc) are necessary. For calculating $pc_{1}$, all data bits and parity bits whose binary representations have a one in the first position are XOR-ed, and so on. In general, for calculating $pc_{i}$, all data bits whose binary representations have a one in the *i*-th position are XOR-ed. The four pc bits indicate the presence or absence of errors. If the four pc bits have a value of zero, there is no error. Otherwise, there is an error in the position obtained, such that $pc_{4}pc_{3}pc_{2}pc_{1}$. In the developed example, the obtained value in binary was 0111; then, the bit in position seven has an error. As last step, the process is finalized when a operation not corrects the error.

If there is no error, the HCs will have a pc value of zero, indicating no error; for TMR, since the three inputs are equal, the output can only have the correct value.

If there is more than one error, HCs cannot detect them, since the pc bits indicate only one position. For TMR, if there are errors in at least two modules (for example, in $\theta_{1}$ and $\theta_{2}$), the three outputs $out\theta_{1}$, $out\theta_{2}$, and $out\theta_{3}$ are different; thus, the voting system is unable to detect a majority output.

The communication channel of VANET is highly dynamic due to the mobility of nodes, the frequent topology change, and the high variability in nodes’ density and neighborhood. Therefore, packet loss estimation impacts the transmission quality of VANETs. The link quality is affected by buildings, trees, road surfaces, and even the weather. Having a good knowledge of the VANET link is vital for designing the upper-layer protocols. According to Jian et al. [48], the node-to-node distance impacts the quality of the link in VANET. There are several error sources, for example, the packet error sequence may be represented as a binary sequence Xk where Xk = 1 if the kth packet is in error and Xk = 0 otherwise. The node-to-node distance impacts the link quality of VANET. The packet error rates will increase with the distance between nodes, and the range of the packet error rate also increases with distance. If the distance is less than 20 m, the packet loss rate will be under 0.05, and it will become larger obviously if the distance is more than 30. The link qualities at different times are more or less similar. The link quality does not vary with time. Whether the car traffic is heavy or light, the packet error rates under the same scenario share the same probability distribution if the node-to-node distance is in the same range. To estimate the packet error rate at the wireless link of VANET, we can use two methods. The first is a passive measurement method based on PLM (packet-level Makov). The second is RPEE (real-time packet error estimation). For further reference to these two methods, we can consult [48].

### 5.3. Incremental Costs for SHA-3 Architectures with and without Fault Tolerance

It is crucial to highlight that Table 6 serves to make two types of comparisons: (A) comparisons between our architecture without fault tolerance and the fault-tolerant architecture, which evaluates within the same design’s ideas, and the advantages and disadvantages that tolerant architectures entail and (B) comparisons between our architectures and related work to understand the contribution by grouping and comparing for two cases, tolerant architectures and nontolerant architectures. If there were no results from the nontolerant architecture of type A, it would be challenging to compare fault-tolerant works because there are no previous works. In this way, comparing our architectures ArchSM and ArchMM against our architectures ArchHC and ArchTMR_HC, there is an increase in hardware resources due to the number of modules based on Hamming Codes and Triple-Modular Redundancy, as well as an increase in latency and minimum period and consequently a decrease in the maximum operating frequency, throughput, and efficiency, although solving faults at different levels. In type B, Table 6 shows that comparing only nontolerant architectures, case A, our architecture reports high performance and high efficiency against related nontolerant architectures, so this design presents an important proposal within this group of ideas. Comparing only our tolerant architectures against tolerant related work, case B, we have the highest performance operating at a lower frequency of operation, improving both time and space complexities.

In Table 6, we show the incremental costs for architectures ArchHC and ArchTMR_HC, taking ArchSM and ArchMM as references, where they are measured in terms of throughput, the cost of adding five step-mapping functions as different modules in ArchMM, Hamming Encoders and Decoders in ArchHC, and Hamming Encoders and Decoders and triplication of step-mapping functions in ArchTMR_HC.

We take as a base the ArchSM architecture for measuring the number of bits that architectures with fault tolerance can process. Thus, it is necessary to measure the throughput of architectures ArchSM, ArchHC, and ArchTMR_HC; consequently, the performance degradation is calculated using a rule of three. The architecture ArchSM implements the five step-mapping functions into one single module and takes one clock cycle for completing one run of Round. ArchHC implements every step-mapping function as a different module and introduces HCs by developing an Encoder and a Decoder for protecting the registers. Some clock cycles are necessary by the Encoder for calculating parity bits at positions that are a power of two. In contrast, extra clock cycles are necessary by the Decoder for recovering the original vector and correcting if there is one error in any position. This implementation increases every run of Round to 12 clock cycles. Therefore, the throughput decreases to a maximum of 245.57 Mbps, corresponding to a performance degradation of 98.18% for the hash size of 224 bits; we show these results in Table 6a. ArchTMR_HC is an improvement of ArchHC. ArchTMR_HC also implements each step-mapping function as a different module and HCs as an Encoder and a Decoder; nevertheless, for achieving higher robustness, a TMR was applied by triplicating every step-mapping function. TMR requires extra clock cycles to determine the majority output using the voting system; then, every run of Round takes 18 clock cycles; consequently, the throughput of ArchTMR_HC decreases to a maximum of 229.95 Mbps, which corresponds to a performance degradation of 98.33% for the hash size of 384 bits. Table 6b shows these results.

In Table 6b, ArchMM is the base for measuring the number of bits that architectures with fault tolerance can process. ArchMM implements every step-mapping as a different module and takes eight clock cycles to complete one run of Round; in addition, it is the basis for forming ArchHC. Since the latter implements HCs, the degradation reaches a throughput of 219.92 Mbps with a performance degradation of 72.65% for the hash size of 256 bits. Similarly, the extra clock cycles necessary for the voting system in ArchTMR_HC decrease the throughput to a maximum of 213.01 Mbps with a performance degradation of 73.50% for the hash size of 256 bits.

The architecture without fault tolerance (ArchSM) can process a maximum of 13,852.09 Mbps for the hash size of 384 bits. Hence, we recommend this architecture for applications requiring considerable processing data with noise-free ambient conditions to provide integrity and authenticity. In contrast, we recommend the two architectures with fault tolerance (ArchHC and ArchTMR_HC) when it is more important to give preference to the protection for generating the hash in the presence of noisy environments, since the change in one single bit can generate a completely different hash due to the avalanche effect.

### 5.4. Error Coverage Capacity for SHA-3 Architectures with Fault Tolerance

**Figure 10:**
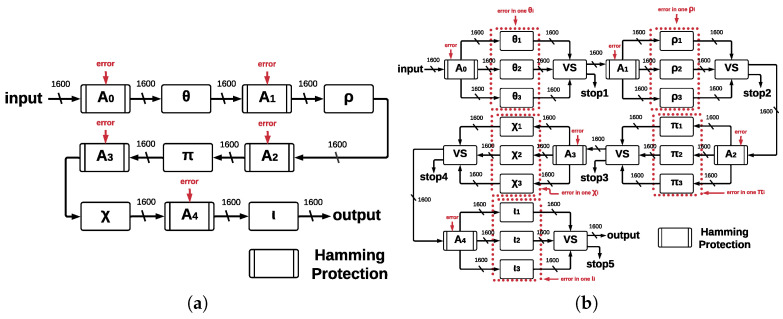
Error injection for ArchHC and ArchTMR_HC. (**a**) Error injection in ArchHC at Round level. (**b**) Error injection in ArchTMR_HC at Round level.

Error coverage capacity shows the number of bits that architectures ArchHC and ArchTMR_HC can handle, in this case, error detection and correction capacity, where ArchHC is based on HCs and ArchTMR_HC on HCs and TMR. HCs’ algorithm can detect and correct one error in any bit; therefore, the developed architectures are limited by the HCs’ capacity. Figure 8a and Figure 9a show the proposed architectures with their five main functions in a single run, operating concurrently and allowing us to implement Hamming protection against errors. If 24 runs are required, then up to 120 errors can be reviewed (this is the case for *ArchHC*). Each of the five central registers has Hamming protection, which involves one implementation of the HCs for every register. There is a maximum of one error that can be detected and corrected for each register (named $A_{1}$, $A_{2}$, $A_{3}$, $A_{4}$, and $A_{5}$), for each run of Round, and KECCAK-p has 24 runs of Round. We remark that it is possible that the SHA-3 algorithm can have *n* runs of KECCAK-p, depending on the size of the message; thus, the total possible number of errors is represented by Equation (2).

(2) $$\begin{array}{c} E=\sum_{k=0}^{n-1}\sum_{j=0}^{23}\sum_{i=0}^{4}e_{ijk}\;\mathrm{such}\;\mathrm{that}\;0\leq i<5,\;\;\;\;\;\;\;\;\;\;\;\;\;\;\;\;\;\;\;\;\;\;\;\;\;\;\;\;\\ \;0\leq j<24,\;0\leq k<n,\;0\leq E\leq 120,\;e:\{0,1\} \end{array}$$

where *E* is the total number of errors in the registers, $e_{ijk}$ is the error in the *i*-th register at the *j*-th run of Round for the k-th run of KECCAK-p. Thus, $e_{ijk}$ can take the value zero if there is no error and one if there is an error. The worst scenario that the architecture ArchHC can afford is the case when each register has one error. In Figure 10a, we represented this scenario; however, if any error occurs in the step-mapping modules, they cannot be detected or corrected, leading to the development of ArchTMR_HC.

Architecture ArchTMR_HC is an improvement of architecture ArchHC since both implement HCs. ArchTMR_HC also can detect and correct a maximum of one error for each register. ArchTMR_HC also implements TMR for the step-mapping functions, allowing the architecture to continue operating if there is one error in one of the three replicas for each step-mapping function (θ, ρ, π, χ, and ι), as shown in Figure 10b. Nevertheless, if the three inputs are different, we cannot obtain a correct output, and a *stop* flag is activated. Hamming protection can detect and correct errors in the five central registers, and TMR allows for operating correctly in the presence of errors at the step-mapping level.

On the one hand, ArchHC error coverage capacity in the worst case is one error in every register. Therefore, since there are five registers, a total of five errors can be detected and corrected for every run of Round; consequently, since KECCAK-p consists of 24 runs by Round. There are a total of 120 errors that can be detected and corrected in the worst case. On the other hand, ArchTMR_HC error coverage capacity in the worst case is one error in every register and one error in every step-mapping function. Hence, since there are five registers and five step-mapping functions, a total of ten errors per every run of Round can be detected and corrected, for a cumulative total of 240 errors for every run of KECCAK-p.

**Figure 11:**
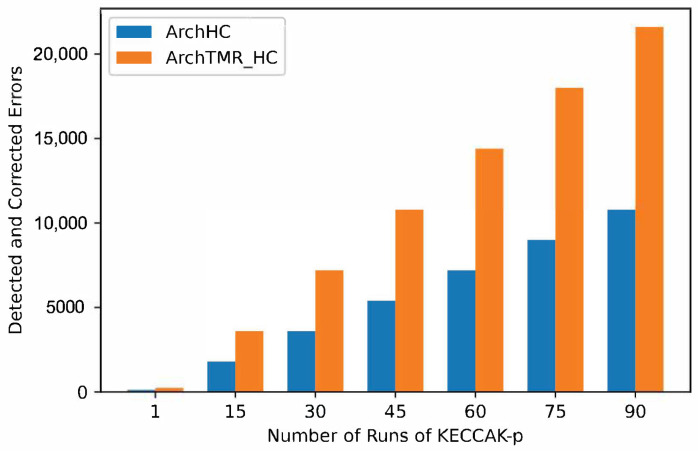
Error coverage capacity.

In Figure 11, the number of detected and corrected errors for every run of KECCAK-p for architectures with fault tolerance is shown, where ArchTMR_HC duplicates the ArchHC capacity. If two KECCAK-p runs are necessary, each architecture duplicates its capacity. If three runs are required, each architecture triplicates its capacity, extending this behavior to any number of runs of KECCAK-p.

### 5.5. Comparison with Other Works

In Table 7, we compare our architectures with other works. We consider architectures without fault tolerance and error detection capacity and with error detection and correction capacity. To the best of our knowledge, the only SHA-3 architectures that can detect and correct errors in the state of the art are ArchHC and ArchTMR_HC. For this reason, no fair comparison with Luo [20] or Bayat [21] can be made, since these last two only can detect errors but not correct them. Nevertheless, Table 7 allows us to illustrate how the techniques of HC and TMR have been implemented in other contexts, identifying the space and time complexity of each work.

The highest throughput and lowest latency in architectures without fault tolerance was achieved by Moumni [49], with a maximum of 33,350 Mbps and two clock cycles, both in the hash size 224; they also reported a throughput of 9930 Mbps and a latency of 24 in the hash size 512, being overcome by the same hash size 512 by ArchSM, which achieved a throughput of 13,667 Mbps. For architectures with error detection, Luo et al. [20] and Bayat et al. [21] did not report the latency. For architectures with error detection and correction, ArchHC achieved a latency of 229 due to HCs and ArchTMR_HC a latency of 443 since HCs and TMR were applied. Other authors did not report the number of LUTs and FFs; however, for architectures without fault tolerance in the proposed work, the numbers of LUTs and FFs are less than those of architectures with error detection and correction. The area was reported only by Luo et al. [20] and Bayat et al. [21] (architectures with error detection), with a better result achieved by Bayat et al. [21] (using an ASIC implementation with an area of 69.249 um${}^{2}$). The minimum period for the proposed work is less in architectures without fault tolerance than in architectures with error detection and correction capacity. The previous result was expected since the number of processes such as HCs and TMR adds complexity to the system; Luo et al. [20] reported a minimum period of 4.5 ns, bigger than proposed architectures without fault tolerance. The maximum achieved frequency is 1192 MHz by Bayat et al. [21] in architectures for error detection, and, similar to minimum period, the frequency results are better in architectures without fault tolerance (reaching a maximum of 233.75 MHz in ArchSM for proposed architectures and a value of 309.6 MHz by Gangwar et al. [50]) than in architectures with error detection and correction capacity, where the maximum is 64.96 MHz in ArchTMR_HC for the hash size of 512 bits. Two different kinds of efficiency were reported, Mbps/LUT (called Efficiency 1) by the proposed work and Mpbs/Slice (called Efficiency 2) by Moumni et al. [49] and Gangwar et al. [50]. For Efficiency 1, higher results were obtained by architectures without fault tolerance than by architectures with error detection and correction capacity. For Efficiency 2, the higher result was obtained by Moumni et al. [49] in the hash size of 224 bits with a value of 13.87 Mbps/Slice.

We remark that if rerunning is executed, then the approach named Time Redundancy is applied, which requires additional hardware for storing intermediate results and logic circuits for selecting a correct response, if the latter exists. Additionally, the critical path can be longer due to store and select output. This idea must be implemented to obtain performance results, make fair comparisons, and avoid assumptions. Another idea for future work, which authors had not considered, is to create an automatic process for rerun until a fault-free result is obtained. The implementation of this idea is not obvious nor direct, since we must consider controversies, such as what to do if there is no convergence to a fault-free result, creating a work queue and how much to store while the corrections are applied, what costs are regarding the temporal and spatial complexities (more hardware resources and larger latency are necessary), the hardware architecture being very different, and that specialized modules must be created, among other situations. These problems can be new research work for a new article.

## 6. Conclusions

Noise or interference might appear in real applications such as automotives (mobile system navigation in diverse environments), Industry 4.0 (a significant amount of machinery, control, and power electronics), and IoT (too many devices communicating on multiple networks), among others, and they can affect cryptographic services because if one error occurs in just a single bit at the input or internal modules, the output will be completely different due to the diffusion, leading to failures of integrity and authentication. The SHA-3 algorithm has advantages for hardware design, security, and flexibility, and it can help to provide security properties such as integrity and authentication.

For these reasons, we implemented two hardware architectures with fault tolerance, which directly contribute to the state of the art. To the best of our knowledge, there are no hardware architectures that can detect and correct errors for the SHA-3 implementations. ArchHC and ArchTMR_HC, can detect and correct (for every run of KECCAK-p) 120 and 240 errors, respectively. Thus, ArchTMR_HC has double the capacity as ArchHC for error correction and detection since a TMR scheme was implemented in the five step-mapping functions. On the other hand, we implemented two architectures without fault tolerance for fair comparisons and trade-off analysis, using them as base references to measure their resources and performances versus our architectures with fault tolerance. We demonstrated that tolerant architectures require more hardware resources, causing more power consumption and higher latency and providing an advantage: fault-tolerant transmission.

Nowadays, several applications and solutions are based on hash algorithms, such as authentication of safety critical messages in VANET [51] or blockchain-assisted authentication schemes to provide message authentication, privacy-preserving, and delay solutions for vehicular networks [52], where SHA-3 is an alternative. The analysis on effects of the faults of the SHA-3 algorithm have been analyzed and modeled from diverse perspectives, for example, Ref. [53] explored attacks for evaluating fault injections and revealing internal states, providing how this injection affects the hash computation and obtaining bounds.

The proposed SHA-3 fault-tolerant hardware architectures (ArchHC and ArchTMR_HC) are a transparent solution for VANET applications. In this sense, both architectures can be part of a greater system to provide integrity, authentication, and digital certificates and be part of a blockchain solution when undesirable conditions can generate and inject faults. The model propagation for VANETs can be affected by outdoor contexts such as climatic elements, environments, terrain factors, buildings, noises occurring by motors, integrated circuits operating at high frequencies, parasitic inductance, capacitance, multiple-transmitting communication networks, among others. One altered bit can change the final hash, causing a failure to authenticate or check integrity, which is critical in automotive applications due to negating resources or access.

We obtained two main results concerning the trade-off of the SHA-3 fault-tolerant hardware architectures (ArchHC and ArchTMR_HC). The first one is related to the throughput measure, where our best results were provided by the ArchHC architecture for the SHA-3 algorithm, which can be used on applications that require at most 276.14 Mbps, focusing on a transparent solution for the user and the system, delivering the hash with a fault-tolerant scheme with a higher cost of hardware resources but solving a high number of faults through TMR and HC. The hardware architecture can not be directly compared with another state-of-the-art FPGA implementation in fault-tolerant contexts, and it can only be unfairly compared with an ASIC architecture [21], where the performance exceeds the 512-bit ArchHC by an order of 92.02 times, working with a frequency of clock that beats us by an order of 23.1 times, which is only used for reference. The second one considers fault tolerance as a measure of performance. In this case, ArchTMR_HC architecture detects and corrects 50% more errors than ArchHC. Therefore, when an architecture robust against errors is needed, ArchTMR_HC should be the choice, although its throughput is lower by 14.85%, since ArchHC reports 276 Mbps and ArchTMR_HC reports 235 Mbps, that is, a difference of 41 Mbps.

Future work may be focused on examining alternatives to HC, such as Hsiao codes, SEC, SEC-DED, OLS (Orthogonal Latin Square), Reed–Muller (RM), Reed–Solomon (RS), and BCH (Bose–Chaudhuri–Hocquenghem). The analysis of these alternatives could reduce the complexity and, consequently, critical path, improving performance. In addition, a prominent result would be to obtain a measure of the impact of implementing the proposed architectures over a VANET scenario, analyzing the functional behavior at the subsystem and system levels.

## Figures and Tables

**Table 1:** Comparison with Related Work.

Work	Algorithm	Application	Techniques	Implementation	Results
This work	SHA-3	authentication in noisy environments	HC and TMR	Virtex-7 FPGA	Throughput: 234.63 Mbps
Luo et al. [20]	SHA-3	protection against faults	parity checking	NanGate FreePDK45	Area: 52,867 um${}^{2}$
Bayat et al. [21]	SHA-3	protection against faults	rotated operands	ASIC	Area: 692.24 um${}^{2}$
Juliato and Gebotys [22]	SHA-256	security in satellites	HC and TMR	Altera Cyclone II	Area: 6232 LEs
Michail et al. [23]	SHA1 SHA-256	security for protocols SET, PKI, IPSec, and VPN	parity codes and hardware redundancy	ASIC	Area: 209,624 um${}^{2}$

**Table 2:** Round and Round Constants algorithms.

0	0000000000000001	12	000000008000808B
1	0000000000008082	13	800000000000008B
2	800000000000808A	14	8000000000008089
3	8000000080008000	15	8000000000008003
4	000000000000808B	16	8000000000008002
5	0000000080000001	17	8000000000000080
6	8000000080008081	18	000000000000800A
7	8000000000008009	19	800000008000000A
8	000000000000008A	20	8000000080008081
9	0000000000000088	21	8000000000008080
10	0000000080008009	22	0000000080000001
11	000000008000000A	23	8000000080008008

**Table 3:** Results comparison of SHA-3 hardware architectures without and with fault tolerance.

Architecture	Hash	Latency	LUT	FF	Minimum Period (ns)	Max. Frequency (Mhz)	Throughput (Mbps)	Efficiency (Mbps/LUT)
ArchSM	224	27	2339	2361	4.38	228.25	13,526	5.78
	256	27	2453	2457	5.14	194.32	11,516	4.69
	384	27	2346	2841	4.27	233.75	13,852	5.90
	512	27	2332	3225	4.33	230.62	13,667	5.86
ArchMM	224	199	2947	10,124	10.47	95.46	768	0.26
	256	199	2947	10,188	9.99	100.01	804	0.27
	384	199	2947	10,444	10.43	95.80	770	0.26
	512	199	2947	10,700	10.51	95.10	765	0.25
ArchHC	224	299	28,703	18,192	21.79	45.89	246	0.0085
	256	299	28,702	18,256	24.33	41.09	220	0.0076
	384	299	28,695	18,512	20.85	47.95	257	0.0089
	512	299	27,224	18,768	19.37	51.60	276	0.010
ArchTMR_HC	224	443	27,226	26,197	15.72	63.58	230	0.0084
	256	443	27,233	26,261	16.95	58.97	213	0.007
	384	443	27,244	26,517	15.70	63.66	230	0.0084
	512	443	27,222	26,773	15.39	64.96	235	0.0086

**Table 4:** Error detection and correction algorithms execution.

(1) First seven bits are considered	
	m = 1001010
(2) Four parity bits are added using the Hamming Encoder (Table 5a)	
	$p_{1}$ = 1 ⊕ 0 ⊕ 1 ⊕ 1 ⊕ 0, $p_{1}$ = 1 $p_{2}$ = 1 ⊕ 0 ⊕ 1 ⊕ 0 ⊕ 0, $p_{2}$ = 0 $p_{3}$ = 1 ⊕ 0 ⊕ 1, $p_{3}$ = 0 $p_{4}$ = 1 ⊕ 0 ⊕ 0, $p_{4}$ = 1 $m^{\prime }$ = 10011010001
(3) One error is generated in a random bit	
	$m^{\prime }$ = 10010010001
(4) The error is detected and corrected when the data are transmitted through the Hamming Decoder	
	$pc_{1}$ = 1 ⊕ 0 ⊕ 0 ⊕ 1 ⊕ 0 ⊕ 1, $pc_{1}=1$ $pc_{2}$ = 1 ⊕ 0 ⊕ 0 ⊕ 0 ⊕ 0 ⊕ 0, $pc_{2}=1$ $pc_{3}$ = 0 ⊕ 0 ⊕ 1 ⊕ 0, $pc_{3}=1$ $pc_{4}$ = 1 ⊕ 0 ⊕ 0 ⊕ 1, $pc_{4}=0$ The error is detected at position seven (0111); a not instruction corrects the error $m^{\prime }$ = 10011010001
(5) The original message *m* at the output of the decoder is transmitted to $\theta_{1}$, $\theta_{2}$, and $\theta_{3}$ $in\theta_{1}$ = 1001010 $in\theta_{2}$ = 1001010 $in\theta_{3}$ = 1001010	
(6) Errors are generated in a random module *i*, for example, $\theta_{1}$	
	$out\theta_{1}$ = 1010000 $out\theta_{2}$ = 0001110 $out\theta_{3}$ = 0001110
(7) The voting system determines the output by a majority vote	
	outputVS = out$\theta_{1}$ × out$\theta_{2}$ + out$\theta_{1}$ × out$\theta_{3}$ + out$\theta_{2}$ × out$\theta_{3}$, outputVS = 0001110

**Table 5:** Hamming Encoder and Decoder processes for error detection and correction in the example of seven bits.

(**a**) Hamming Encoder process for the example of seven bits												
	$d_{7}$	$d_{6}$	$d_{5}$	$p_{4}$	$d_{4}$	$d_{3}$	$d_{2}$	$p_{3}$	$d_{1}$	$p_{2}$	$p_{1}$	
position	1011	1010	1001	1000	0111	0110	0101	0100	0011	0010	0001	
original word	1	0	0		1	0	1		0			
$p_{1}$	1		0		1		1		0		1	
$p_{2}$	1	0			1	0			0	0		
$p_{3}$					1	0	1	0				
$p_{4}$	1	0	0	1								
original + parity	1	0	0	1	1	0	1	0	0	0	1	
(**b**) Hamming Decoder process for the example of seven bits with one error												
	$d_{7}$	$d_{6}$	$d_{5}$	$p_{4}$	$d_{4}$	$d_{3}$	$d_{2}$	$p_{3}$	$d_{1}$	$p_{2}$	$p_{1}$	parity check
position	1011	1010	1001	1000	0111	0110	0101	0100	0011	0010	0001	
original + parity	1	0	0	1	0	0	1	0	0	0	1	
$pc_{1}$	1		0		0		1		0		1	1
$pc_{2}$	1	0			0	0			0	0		1
$pc_{3}$					0	0	1	0				1
$pc_{4}$	1	0	0	1								0

**Table 6:** Incremental costs.

(**a**) Taking ArchSM as a basis			
Architecture/Hash		Throughput (Mbps)	Performance Degradation (%)
ArchSM	224	13,526.42	-
	256	11,515.59	-
	384	13,852.09	-
	512	13,666.80	-
ArchHC	224	245.57	98.18
	256	219.92	98.09
	384	256.62	98.14
	512	276.14	97.97
ArchTMR_HC	224	229.66	98.30
	256	213.01	98.15
	384	229.95	98.33
	512	234.63	98.28
(**b**) Taking ArchMM as a basis			
Architecture/Hash		Throughput (Mbps)	Performance Degradation (%)
ArchMM	224	767.56	-
	256	804.10	-
	384	770. 28	-
	512	764.64	-
ArchHC	224	245.57	68
	256	219.92	72.65
	384	256.62	66.68
	512	276.14	63.88
ArchTMR_HC	224	229.66	70.07
	256	213.01	73.50
	384	229.95	70.14
	512	234.63	69.31

**Table 7:** Results comparison among different architectures.

	Design	Hash	Latency	LUT’s	FF	Area (um${}^{2}$)	Timing (ns)	Frequency (Mhz)	Throughput (Mbps)	Efficiency 1 (Mbps/LUT)	Efficiency 2 (Mbps/Slice)
Without Fault Tolerance	ArchSM	224	27	2339	2361	-	4.38	228.25	13,526	5.78	-
		256	27	2453	2457	-	5.14	194.32	11,516	4.69	–
		384	27	2346	2841	-	4.27	233.75	13,852	5.90	-
		512	27	2332	3225	-	4.33	230.62	13,667	5.86	-
	ArchMM	224	199	2947	10,124	-	10.47	95.46	768	0.26	-
		256	199	2947	10,188	-	9.99	100.01	804	0.27	-
		384	199	2947	10,444	-	10.43	95.80	770	0.26	-
		512	199	2947	10,700	-	10.51	95.10	765	0.25	-
	Moumni [49]	224	24	-	-	-	-	-	19,860	-	13.87
		256	24	-	-	-	-	-	18,750	-	13.10
		384	24	-	-	-	-	-	14,340	-	10.02
		512	24	-	-	-	-	-	9,930	-	6.93
		224	2	-	-	-	-	-	33,350	-	2.14
		256	2	-	-	-	-	-	31,500	-	2.02
		384	2	-	-	-	-	-	24,090	-	1.55
		512	2	-	-	-	-	-	16,670	-	1.07
	Gangwar [50]	-	24	-	-	-	-	309.6	14,040	-	11.24
Error Detection	Luo [20]	-	-	-	-	52,867.2	4.5	-	-	-	-
	Bayat [21]	-	-	-	-	69.24	-	1192	25,400	-	-
Error Detection and Correction	ArchHC	224	299	28,703	18,192	-	21.79	45.89	246	0.008555	-
		256	299	28,702	18,256	-	24.33	41.09	220	0.0076	-
		384	299	28,695	18,512	-	20.85	47.95	257	0.0089	-
		512	299	27,224	18,768	-	19.37	51.60	276	0.010	-
	ArchTMR_HC	224	443	27,226	26,197	-	15.72	63.58	230	0.0084	-
		256	443	27,233	26,261	-	16.95	58.97	213	0.007	-
		384	443	27,244	26,517	-	15.70	63.66	230	0.0084	-
		512	443	27,222	26,773	-	15.39	64.96	235	0.0086	-

## Abbreviations

The following abbreviations are used in this manuscript:

ArchHC	Architecture with fault tolerance based on HC
ArchMM	Architecture with Multiple Modules
ArchSM	Architecture with a Single Module
ArchTMR_HC	Architecture with fault tolerance based on HCs and TMR
Constants r[x,y]	rotation constants
Constants RC	Round constants
E	number of errors
ECU	engine control unit
FPGA	Field-Programmable Gate Arrays
Function pad	padding
Functions θ, ρ, π, χ, ι	main functions of SHA-3
HC	Hamming Codes
I4.0	Industry 4.0
IIoT	Industrial Internet Of Things
IoT	Internet Of Things
LUT	look-up table
MAC	message authentication code
MAC layer	medium-access control layer
MD4	message-digest algorithm 4
MD5	message-digest algorithm 5
Operator ⊕	XOR operation
Operator ROT(W, r)	rotation of W by r times
RSU	roadside units
SDRAM	static random-access memory
SHA	Secure Hash Algorithm
TMR	Triple-Modular Redundancy
VANET	vehicular ad hoc network
V2I	vehicle-to-infrastructure
V2V	vehicle-to-vehicle
Variable d	output size
Variable c	capacity
Variable M	message
Variable N	input bit string
Variable r	rate or block size
